# Diagnosis of Pulmonary Hypertension with Cardiac MRI: Derivation and Validation of Regression Models

**DOI:** 10.1148/radiol.2018180603

**Published:** 2018-10-23

**Authors:** Christopher S. Johns, David G. Kiely, Smitha Rajaram, Catherine Hill, Steven Thomas, Kavitasagary Karunasaagarar, Pankaj Garg, Neil Hamilton, Roshni Solanki, David A. Capener, Charles Elliot, Ian Sabroe, Athanasios Charalamopopoulos, Robin Condliffe, James M. Wild, Andrew J. Swift

**Affiliations:** From the Academic Department of Radiology, University of Sheffield, Floor C, Royal Hallamshire Hospital, Glossop Rd, Sheffield S10 2JF, England (C.S.J., P.G., R.S., D.A.C., J.M.W., A.J.S.); and Sheffield Pulmonary Vascular Disease Unit (D.G.K., S.R., N.H., C.E., I.S., A.C., R.C.) and Department of Radiology (C.S.J., C.H., S.T., K.K., A.J.S.), Sheffield Teaching Hospitals, Sheffield, England.

## Abstract

**Purpose:**

To derive and test multiparametric cardiac MRI models for the diagnosis of pulmonary hypertension (PH).

**Materials and Methods:**

Images and patient data from consecutive patients suspected of having PH who underwent cardiac MRI and right-sided heart catheterization (RHC) between 2012 and 2016 were retrospectively reviewed. Of 2437 MR images identified, 603 fit the inclusion criteria. The mean patient age was 61 years (range, 18–88 years; mean age of women, 60 years [range, 18–84 years]; mean age of men, 62 years [range, 22–88 years]). In the first 300 patients (derivation cohort), cardiac MRI metrics that showed correlation with mean pulmonary arterial pressure (mPAP) were used to create a regression algorithm. The performance of the model was assessed in the 303-patient validation cohort by using receiver operating characteristic (ROC) and χ^2^ analysis.

**Results:**

In the derivation cohort, cardiac MRI mPAP model 1 (right ventricle and black blood) was defined as follows: −179 + log_e_ interventricular septal angle × 42.7 + log_10_ ventricular mass index (right ventricular mass/left ventricular mass) × 7.57 + black blood slow flow score × 3.39. In the validation cohort, cardiac MRI mPAP model 1 had strong agreement with RHC-measured mPAP, an intraclass coefficient of 0.78, and high diagnostic accuracy (area under the ROC curve = 0.95; 95% confidence interval [CI]: 0.93, 0.98). The threshold of at least 25 mm Hg had a sensitivity of 93% (95% CI: 89%, 96%), specificity of 79% (95% CI: 65%, 89%), positive predictive value of 96% (95% CI: 93%, 98%), and negative predictive value of 67% (95% CI: 53%, 78%) in the validation cohort. A second model, cardiac MRI mPAP model 2 (right ventricle pulmonary artery), which excludes the black blood flow score, had equivalent diagnostic accuracy (ROC difference: *P* = .24).

**Conclusion:**

Multiparametric cardiac MRI models have high diagnostic accuracy in patients suspected of having pulmonary hypertension.

Published under a CC BY 4.0 license.

*Online supplemental material is available for this article.*

See also the editorial by Colletti in this issue.

**Summary**

A cardiac MRI diagnostic model for the identification of pulmonary hypertension, incorporating measurements from the right ventricle and pulmonary artery, accurately correlates with right-sided heart catheterization–measured mean pulmonary arterial pressure and represents a noninvasive method to assess the diagnosis of pulmonary hypertension.

**Implications for Patient Care**

■ The mean pulmonary arterial pressure determined with a cardiac MRI model that includes the angle of the interventricular septum, ventricular mass index, and the extent of black blood slow flow correlated with that measured with right-sided heart catheterization.
■ Cardiac MRI parameters can help identify patients with high sensitivity (93%) and moderate specificity (79%).

## Introduction

Pulmonary hypertension (PH) is a condition with high morbidity and mortality. Regardless of cause, PH has a negative effect on quality of life (1) and is life-shortening, even with modern treatment strategies. Right-sided heart catheterization (RHC) is the reference standard for PH, which is defined as a mean pulmonary arterial pressure (mPAP) of at least 25 mm Hg at rest (2).

RHC is an invasive test with a serious complication rate of 1% (3), so current guidelines recommend echocardiography to classify patients suspected of having PH as having low, medium, or high risk of PH (2). Doppler echocardiographic measurement of systolic pulmonary arterial pressure requires an estimation of right atrial pressure; the current estimation from inferior vena cava size does not enable an accurate prediction of the true right atrial pressure at RHC (4). Echocardiography also requires tricuspid regurgitation, which, while commonly seen in PH, is not always present, and severe tricuspid regurgitation causes erroneous low or high echocardiographic predictions of mPAP (5). As such, estimation of pulmonary arterial pressures from echocardiography shows only moderate agreement with that measured with RHC (6), with 95% limits of agreement at Bland-Altman analysis ranging from +38.8 to −40.0 mm Hg (7). A large meta-analysis showed only modest diagnostic accuracy of echocardiography for the presence of PH, with a sensitivity of 83% and a specificity of 72% (8). Furthermore, echocardiography is not possible in the context of severe lung disease owing to the loss of acoustic windows, and when it is measurable it is often erroneous (9).

A number of cardiac MRI metrics have been proposed as predictors of increased pulmonary arterial pressure, and a number of image-based models (physiologic, empirical, and computational) have been proposed. Black blood scoring has been shown to be a useful diagnostic marker in PH; however, it is not widely adopted in MRI protocols. Thus, developing models with and without this parameter would be advantageous. Previously identified cardiac MRI metrics and markers have been tested in relatively small numbers and often have modest diagnostic accuracy.

We hypothesized that *(a)* cardiac MRI parameters from both the heart and pulmonary arteries have additive diagnostic value in patients suspected of having PH and *(b)* a cardiac MRI multiparametric model could help diagnose PH with high accuracy.

The aim of this study was to derive two regression models, one with and one without black blood scoring, to diagnose PH with use of cardiac and pulmonary vascular cardiac MRI.

## Materials and Methods

### Patients

**Figure 1:**
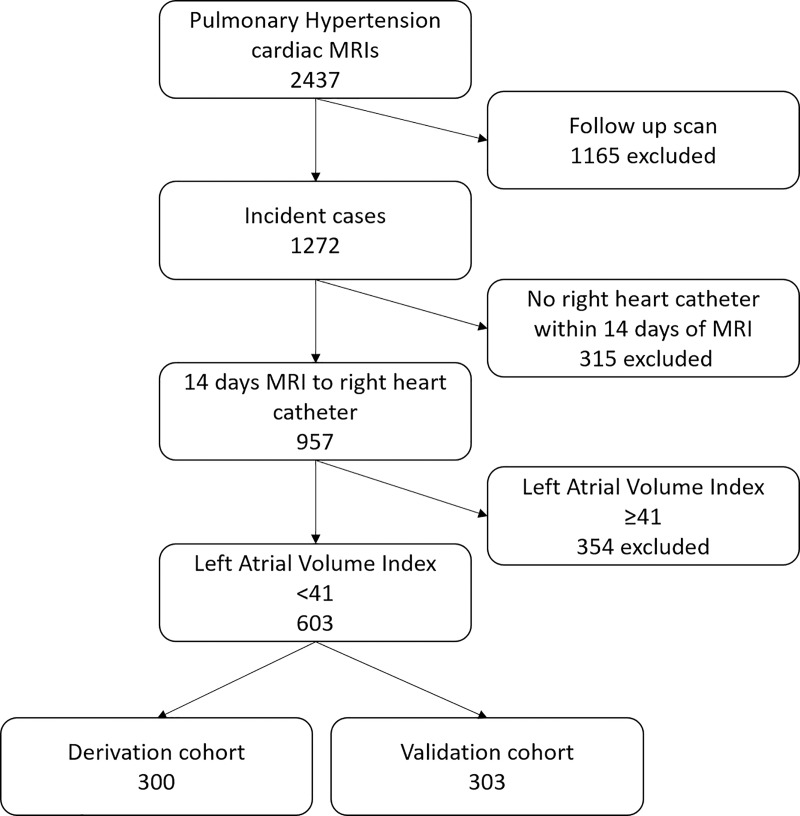
Patient flowchart.

Consecutive incident patients suspected of having PH who were referred to a tertiary PH center from April 2012 to October 2016 were identified from the ASPIRE database (Assessing the Spectrum of Pulmonary Hypertension Identified at a Referral Center) (10). Patients were separated by date of imaging into a derivation (*n* = 300) and a validation (*n* = 303) cohort. Only incident patients who underwent cardiac MRI and RHC within 14 days were included. Any patient with a left atrial volume index of 41 mL or higher at cardiac MRI was excluded as this is a potential marker of PH due to left-sided heart disease (11). Figure 1 shows the process of patient selection. Treatment was always initiated after RHC and cardiac MRI had been performed. Ethical approval was granted from a local ethics committee for this retrospective study, and the requirement to obtain informed written consent was waived (ref c06/Q2308/8).

### Image Acquisition

Cardiac MRI was performed with a 1.5-T whole-body imager (HDx; GE Healthcare, Milwaukee, Wis), with the patient supine, by using an eight-channel cardiac coil. Standard four-chamber and short-axis balanced steady-state free precession cine stack images were acquired from base to apex. Phase contrast imaging was performed orthogonal to the main pulmonary artery. Black blood imaging of the pulmonary arteries was performed by using a double inversion recovery fast spin-echo sequence, acquiring images with 8-mm-thick sections through the main and branch pulmonary arteries (12). The sequence parameters are provided in Appendix E1 (online).

### Image Analysis

MR images were manually analyzed by D.A.C. (an MRI radiographer with 9 years of cardiac experience) with use of an Advantage workstation (version 4.4, GE Healthcare) with ReportCard software (GE Healthcare). Analysis was performed prospectively and sequentially at the time of imaging, with remote supervision by A.J.S. (a consultant cardiac radiologist with 12 years of experience) when required. Image analysis was performed before RHC and while blinded to all other clinical details. The results of the analysis and, frequently, the contours were reviewed at the weekly multidisciplinary meeting, and any studies that were not of diagnostic quality were excluded. All cardiac MRI metrics outlined below were remeasured in 15 randomly selected studies from the validation cohort by a second reviewer (C.S.J., a general radiologist with 6 years of experience) who was blinded to the findings of the previous analysis and to all clinical data.

**Figure 2:**
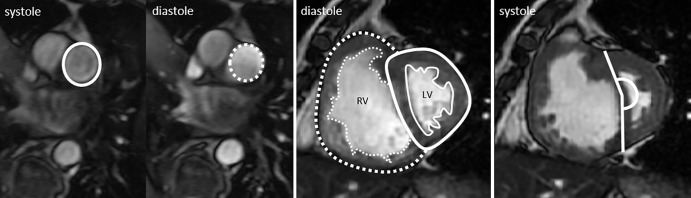
Representative steady-state free precession short-axis cine images of pulmonary artery demonstrate calculation of the quantitative metrics pulmonary arterial relative area (left), with systolic pulmonary artery size shown as solid line and diastolic pulmonary artery size shown as dotted line, ventricular mass index (middle), with right ventricular mass shown as dotted area and left ventricular mass shown as solid lined area, and interventricular septal angle (right), shown by solid line. *LV* = left ventricle, *RV* = right ventricle.

Left and right ventricular end-diastolic volume, end-systolic volumes, and right and left ventricular stroke volume and mass were calculated (indexed to body surface area) and right and left ventricular ejection fraction, ventricular mass index (right ventricular mass divided by left ventricular mass) (13,14), and interventricular septal angle were measured as previously described (15–17). Maximal and minimal pulmonary artery areas were manually traced, and relative area change was defined as follows: pulmonary artery relative area change = (maximum area − minimum area)/minimum area (18,19). Pulmonary artery average velocity was calculated by using the phase contrast flow as forward pulmonary artery velocity/diastolic pulmonary artery size (Fig 2). The reproducibility of these cardiac MRI metrics between the reviewer D.A.C. (MR radiographer) and A.J.S. (consultant cardiac radiologist) has been previously published in a cohort of patients with pulmonary arterial hypertension (group 1) (20).

**Figure 3:**
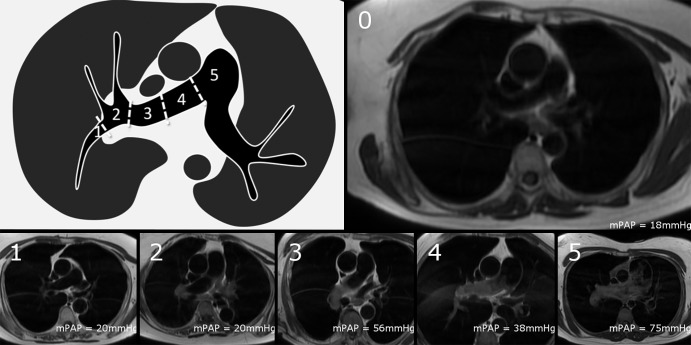
Semiquantitative scoring of black blood slow flow score. The score is based on how proximal signal can be seen in pulmonary arteries, where *0* = no slow flow, *1* = signal within segmental pulmonary arteries, *2* = signal within lobar branches, *3* = signal within distal main pulmonary artery, *4* = signal within proximal main pulmonary artery, and *5* = signal within proximal main pulmonary artery. Diagram demonstrates different parts of pulmonary artery and naming convention. Example images of each score are provided with their mean pulmonary artery pressure (*mPAP*) as a reference.

Black blood slow flow was visually scored by A.J.S. by using a semiquantitative scale from 0 to 5 depending on how proximal the flow feature is seen in the pulmonary arterial tree (Fig 3), as follows: 0 = absent, 1 = segmental, 2 = lobar, 3 = distal main, 4 = proximal main, and 5 = trunk. This scale has been shown to have good interobserver reproducibility (21).

### Right-sided Heart Catheterization

RHC was performed as part of the routine clinical pathway by the PH clinicians (D.G.K., C.E., and R.C.), all with more than 10 years of experience, by using a balloon-tip 7.5-F thermodilution catheter (Becton-Dickinson, Franklin Lakes, NJ). PH was defined as a resting mPAP of 25 mm Hg or higher (2).

### Statistical Analysis

Statistical analysis was performed with software (SPSS 22; IBM, Chicago, Ill), and graphs were produced by using GraphPad Prism (GraphPad Software, La Jolla, Calif). Unless otherwise stated, *P* < .05 was considered indicative of a statistically significant difference. Comparison of continuous variables between groups was made by using the Student *t* test and the χ^2^ test for discrete variables.

***Derivation cohort.—***The first 300 patients were used as a derivation cohort to derive diagnostic PH models. The Pearson correlation coefficients between quantifiable cardiac MRI characteristics and mPAP were calculated. As multiple correlations were assessed, the risk of type 1 error was reduced by using Bonferroni correction, so *P* < .002 was considered indicative of a statistically significant difference (30 different correlations). Scatterplots were constructed to ensure linearity between cardiac MRI metrics and mPAP. Any significant correlations were used to calculate a linear regression equation for estimation of mPAP. A receiver operating characteristic (ROC) curve was constructed to assess suitable diagnostic thresholds, one that was highly specific and one that had a balance of specificity and sensitivity (the Youden index). A second regression equation was calculated without the use of black blood slow flow, and diagnostic accuracy was calculated with the same method.

***Validation cohort.—***The second half of the patients (*n* = 303) was used for validation. The correlation of the derived regression equations for PH diagnosis against RHC-measured mPAP was calculated. The accuracy of these models was assessed with Bland-Altman analysis, and the intraclass correlation coefficient was calculated between the model- and RHC-measured mPAP (two-way mixed effects model, absolute agreement definition, and single measures). Their diagnostic performance was assessed by using ROC curve analysis, sensitivity, specificity, negative and positive predictive values, and negative and positive likelihood ratios. For comparison of ROC curves, we used a nonparametric method that is analogous to the Wilcoxon–Mann-Whitney test. Reproducibility between D.A.C. and C.S.J. was analyzed with the intraclass correlation coefficient by using a two-way mixed effects model and average measures.

## Results

### Patients

The ASPIRE MRI subregistry contains 2437 cardiac MR images, and within this there are 1272 incident images with suspected PH. Of the patients with incident images, 957 underwent RHC and cardiac MRI within 14 days. Of those 957 patients, 603 had a left atrial volume index of less than 41 mL. The first 300 patients were used as the derivation cohort and the second 303 as the validation cohort (Figure 1). Baseline characteristics of the patients are provided in Table 1. Apart from cardiac index, there were no significant differences in the clinical demographics of patients between derivation and validation cohorts (Table 2). The difference in cardiac index between the two groups was very small and unlikely to be of clinical significance, as outlined in Table 1. Fifty-two patients in the derivation cohort and 45 in the validation cohort did not have PH. In total, there were 97 patients who did not have PH, 264 patients with pulmonary arterial hypertension, 11 with PH due to left-sided heart disease (even after exclusion of dilated left atria), 60 with PH due to respiratory disease, 157 with chronic thromboembolic disease, and 18 with PH due to unclear or multifactorial mechanisms. Three hundred fifteen patients were excluded because MRI was performed more than 14 days from RHC. This excluded group consisted of 37 patients without PH, 154 patients with group 1 pulmonary arterial hypertension, 19 with PH due to left-sided heart disease, 16 with PH due to lung disease, 81 with chronic thromboembolic PH, and eight with unclear causes.

**Table 1:**
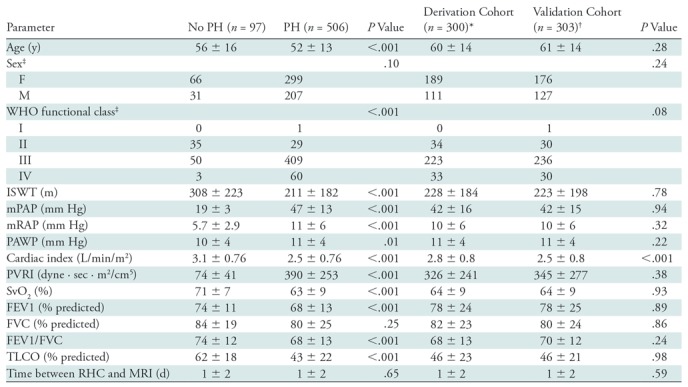
Baseline Demographics for All Patients according to PH Status and Derivation or Validation Cohort

Note.—Except where indicated, data are means ± standard deviations. *P* values were calculated by using the Student *t* test for continuous variables and the χ^2^ test for discrete variables. FEV1 = forced expiratory volume in 1 second, FVC = forced vital capacity, ISWT = incremental shuttle walk test, mPAP = right-sided heart catheterization–measured mean pulmonary arterial pressure, mRAP = mean right atrial pressure, PAWP = pulmonary artery wedge pressure, PH = pulmonary hypertension, PVRI = pulmonary vascular resistance index, RHC = right-sided heart catheterization, SvO_2_ = mixed venous oxygen saturation, TLCO = transfer factor for carbon monoxide, WHO = World Health Organization.

*There were 52 patients without PH and 218 with PH.

^†^There were 45 patients without PH and 258 with PH.

^‡^Data are numbers of patients.

**Table 2:**
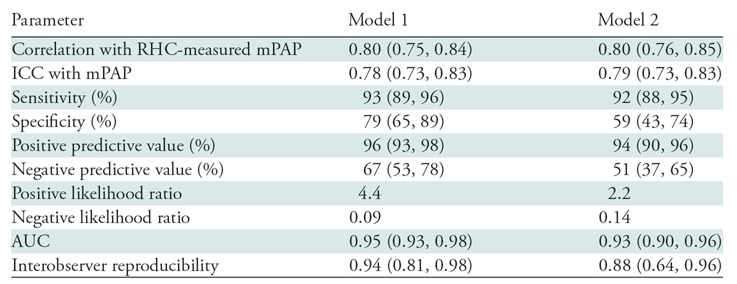
Diagnostic Performance of Models 1 and 2

Note.—Numbers in parentheses are 95% confidence intervals. AUC = area under the receiver operating characteristic curve, ICC = intraclass correlation coefficient, mPAP = mean pulmonary arterial pressure, RHC = right-sided heart catheterization.

### Derivation Cohort

The strongest correlations with mPAP were for systolic interventricular septal angle, black blood score, right ventricular ejection fraction, right ventricular end-systolic volume index, and ventricular mass index (*R* = 0.74, 95% confidence interval [CI]: 0.69, 0.79; *R* = 0.69, 95% CI: 0.63, 0.75; *R* = −0.60, 95% CI: −0.53, 0.67; *R* = 0.59, 95% CI: 0.51, 0.65; and *R* = 0.53, 95% CI: 0.44, 0.61, respectively). Higher right ventricular volumes (end-diastolic and end-systolic volumes), lower left atrial volume index and left ventricular end diastolic volume, larger pulmonary artery size, and lower pulmonary arterial relative area change and velocity all showed statistically significant correlation with higher mPAP (see Table E1 [online] for the data). Visual inspection of the scatterplots of interventricular septal angle and ventricular mass index revealed an exponential relationship, so the log_10_ of the ventricular mass index and log_e_ of septal angle were used in further calculations.

Regression analysis, performed in a forward direction for any metric that showed a correlation with mPAP (*P* < .2), produced the following equation: cardiac MRI mPAP model 1 (right ventricle and black blood) = −179 + log_e_ interventricular septal angle × 42.7 + log_10_ ventricular mass index (right ventricular mass/left ventricular mass) × 7.57 + black blood slow flow score × 3.39.

A threshold of at least 25 mm Hg was identified as having an optimal diagnostic threshold (Youden index), with a sensitivity of 96% and a specificity of 73%.

After exclusion of black blood slow flow, the following regression equation was derived: cardiac MRI mPAP model 2 (right ventricle pulmonary artery) = −231.423 + log_e_ interventricular septal angle × 53.8 + log_10_ ventricular mass index (right ventricular mass/left ventricular mass) × 8.708 + diastolic pulmonary artery area × 0.009. For cardiac MRI mPAP model 2, the threshold of at least 25 mm Hg was again identified as having an optimal diagnostic threshold.

### Validation Cohort

**Figure 4:**
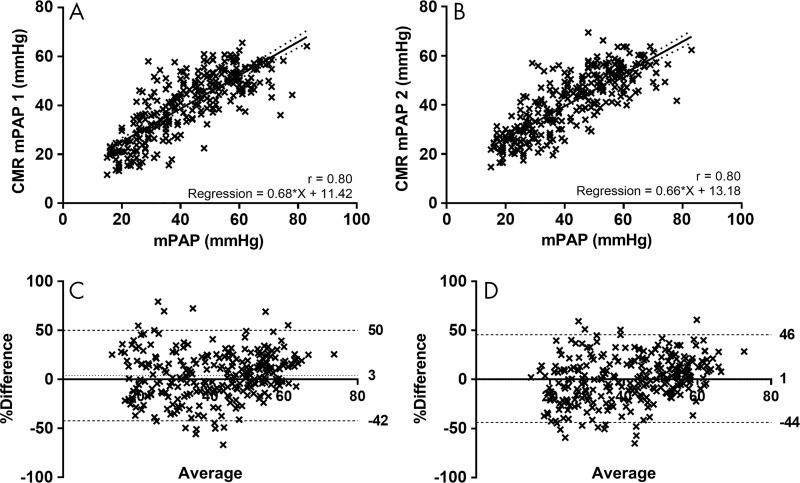
*A, B*, Scatterplots show correlation of, *A*, cardiac MRI *(CMR)* mean pulmonary arterial pressure (mPAP) model 1 (the model based on metrics from right ventricle and slow flow at black blood imaging) and, *B*, cardiac MRI mPAP model 2 (the model based on metrics of the pulmonary artery and right ventricle) with right-sided heart catheterization–measured mPAP in validation cohort. *C, D*, Corresponding Bland-Altman plots. Dotted line shows bias and dashed lines show 95% levels of agreement.

***Agreement.—***Cardiac MRI mPAP model 1 correlated strongly with RHC-measured mPAP (*R* = 0.80; 95% CI: 0.75, 0.84). Bland-Altman analysis showed a small bias (3.9%), with reasonable 95% agreement (−42% to 50%) and an excellent intraclass correlation coefficient for the estimation of mPAP of 0.78 (95% CI 0.73, 0.83). The cardiac MRI mPAP model 2 also strongly correlated with mPAP (*R* = 0.80; 95%: CI: 0.76, 0.85), with a small bias (0.9%) and again reasonable 95% agreement (−43% to 46%), with an intraclass correlation coefficient of 0.79 (95% CI: 0.73, 0.83). Figure 4 shows the scatterplots and Bland-Altman plots for cardiac MRI mPAP models 1 and 2.

***Diagnostic accuracy.—***Cardiac MRI mPAP model 1 showed high diagnostic accuracy (area under the ROC curve = 0.95; 95% CI: 0.93, 0.98). The threshold of at least 25 mm Hg had a sensitivity of 93% (95% CI: 89%, 96%), specificity of 79% (95% CI: 65%, 89%), positive predictive value of 96% (95% CI: 93%, 98%), negative predictive value of 67% (95% CI: 53%, 78%), positive likelihood ratio of 4.4, and negative likelihood ratio of 0.09.

**Figure 5:**
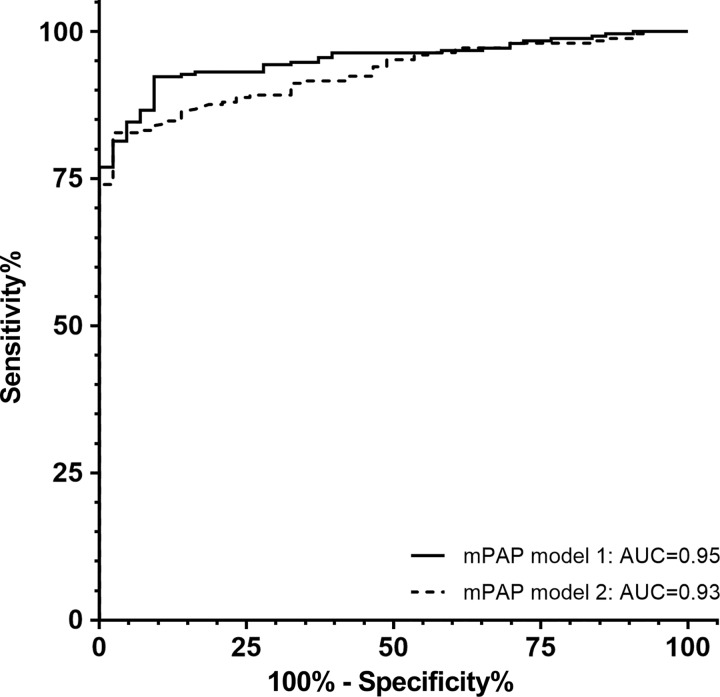
Receiver operating characteristics (ROC) curve for cardiac MRI mean pulmonary arterial pressure (*mPAP*) models 1 and 2. There is no significant difference between the two curves (*P* = .24). *AUC* = area under the ROC curve.

Cardiac MRI mPAP model 2 (≥25 mm Hg) had a sensitivity of 92% (95% CI: 88%, 95%), specificity of 59% (95% CI: 43%, 74%), positive predictive value of 94% (95% CI: 90%, 96%), negative predictive value of 51% (95% CI: 37%, 65%), positive likelihood ratio of 2.2, and negative likelihood ratio of 0.14. ROC curve analysis of cardiac MRI mPAP model 2 showed an area under the ROC curve of 0.93 (95% CI: 0.90, 0.96). There was no significant difference in the area under the ROC curve between these models (*P* = .24). Figure 5 shows the ROC curves for both models, and Table 2 provides a summary of the diagnostic performance of both models.

After exclusion of all patients with PH due to left-sided heart disease, in addition to exclusion of patients with a left atrial volume index of less than 41 mL, cardiac MRI mPAP model 1 had a sensitivity of 94% (95% CI: 91%, 97%), specificity of 79% (95% CI: 65%, 89%), positive predictive value of 96% (95% CI: 93%, 98%), and negative predictive value of 72% (95% CI: 58%, 83%). Cardiac MRI mPAP model 2 had a sensitivity of 94% (95% CI: 91%, 97%), specificity of 51% (95% CI: 37%, 65%), positive predictive value of 92% (95% CI: 88%, 94%), and negative predictive value 61% (95% CI: 45%, 75%).

Interobserver reproducibility analysis showed an intraclass correlation coefficient of 0.94 (95% CI: 0.81, 0.98) for cardiac MRI mPAP model 1 and 0.88 (95% CI: 0.64, 0.96) for cardiac MRI mPAP model 2.

## Discussion

Interventricular septal angle, ventricular mass index, and black blood slow flow score were independent cardiac MRI predictors of mean pulmonary arterial pressure (mPAP), with additive value for the diagnosis of pulmonary hypertension (PH). We have derived a linear regression model, a composite of these three measurements (in 300 patients), and have shown high diagnostic accuracy in a large validation cohort (a further 303 patients), identifying a threshold of at least 25 mm Hg as the optimal threshold, equivalent to that with RHC.

Our proposed cardiac MRI mPAP 1 (right venticle and black blood) model comprises measurement of displacement of the interventricular septum (a marker of the pressure and volume differential between the left and right ventricle), remodeling of the right ventricle (ventricular mass index), and slow or turbulent flow in the pulmonary artery at black blood imaging. These measurements are easily acquired from standard cardiac MRI sequences, require little postprocessing time, and are reproducible (20). Black blood pulmonary arterial flow score is a reproducible marker of outcome in pulmonary arterial hypertension (21). In healthy individuals, the protons in the pulmonary artery that are excited are replaced by nonexcited protons from outside the field of view. In patients with PH, the excited protons within the pulmonary artery remain, returning signal. This is likely to be a composite of slow flow and vortical or turbulent flow within the pulmonary artery. The increasing use of four-dimensional phase-contrast flow imaging will likely allow this metric within the model to be improved upon (22–24). The parameters of black blood imaging that are susceptible to flow artifacts (section thickness, inversion time, echo time, echo train length, and cardiac triggering point) were kept the same between patients and could be easily adopted for standardized use in different centers.

Previous studies have shown that the measurement of septal deviation has diagnostic value in suspected PH (25); in addition, it has been shown previously that the addition of ventricular mass index to a measure of septal angle increased diagnostic accuracy (15). The current larger study further validates these observations and identifies that the addition of a measurement from the pulmonary vasculature increases diagnostic accuracy (ie, black blood slow flow score or pulmonary artery size in diastole). Computational models of pulmonary arterial flow have also been assessed for the diagnosis of PH (26). Further work to integrate such physiologic models would be of value.

In patients with PH due to left-sided heart disease, treatment is aimed at the underlying cause (often left ventricular diastolic dysfunction) and not at the pulmonary vasculature. As such, current guidelines recommend that patients suspected of having PH but who have left-sided heart disease and no features of severe PH should not be referred to specialist centers (2). We have considered this recommendation in our patient selection, using cardiac MRI–derived left atrial volume index (27) (≥41 mL/m^2^) as a cardiac MRI marker for the presence of left-sided heart disease (11). Further work is required to improve the identification of patients with PH due to left-sided heart disease by using cardiac MRI metrics such as transmitral flow and left atrial and ventricular filling.

Our study has some limitations, including its single-center, retrospective design. To further assess the validity of our models, analysis of a further cohort of patients suspected of having PH is required at a different center. In addition, it is necessary to validate the models in a cohort with a relatively lower proportion of patients with PH. The tertiary referral center population potentially limits its applicability in the wider clinical setting. Black blood imaging may not be available in some centers, or sequence parameters may differ between centers. In these instances, cardiac MRI mPAP model 2 (right ventricle pulmonary artery) may be used based on the interventricular septal angle, ventricular mass index, diastolic pulmonary arterial size, and pulmonary arterial relative area change. The limits of agreement of cardiac MRI models are insufficient for accurate estimation of an individual mPAP value. However, diagnostic accuracy is high as a diagnostic test at the threshold of 25 mm Hg.

In conclusion, cardiac MRI has high diagnostic accuracy in a cohort of incident patients referred to a tertiary referral center with suspected pulmonary hypertension (PH). A reproducible model comprising simple and easy-to-obtain metrics (interventricular septal angle, ventricular mass index, and black blood score) can enable the identification of patients with PH with high accuracy. This model may improve the detection rates of PH and enhance the noninvasive assessment of patients with this severe disease.

## APPENDIX

Appendix E1, Table E1 (PDF)
