# Supplementary material for: Diagnosis of Pulmonary Hypertension with Cardiac MRI: Derivation and Validation of Regression Models
Source: Radiology. 2018 Oct 23;290(1):61–8. doi: 10.1148/radiol.2018180603 (PMC6314564; doi:10.1148/radiol.2018180603)
Supplement: Appendix E1, Table E1 (PDF) [file ry180603suppa1.pdf]

Published under a CC BY 4.0 license.
10.1148/radiol.2018180603

Appendix E1

MRI Sequence Parameters

Four-chamber (4Ch) and short axis (SA) cine images were acquired using a retrospectively cardiac gated multislice steady-state free precession (SSFP) sequence, as previously published (12). A stack of axial images in the SA plane with slice thickness of 8 mm with a 2 mm interslice gap or 10 mm with no interslice gap were acquired, covering both ventricles from base to apex. The SSFP sequence parameters were: TR 2.8 ms, TE 1.0 ms, flip angle 50°, 256 × 256 matrix, 125 kHz bandwidth, and slice thickness 8 to 10 mm.

Phase contrast imaging was performed perpendicular to the pulmonary trunk. The phase contrast imaging parameters were: TR 5.6 ms, TE 2.7 ms, slice thickness 10 mm, bandwidth 62.5 kHz, matrix 256 × 128 and velocity encoding 150 cm/s. The images were retrospectively ECG gated with 40 phases. FOV adjusted for body size for all CINE and flow imaging. The PA images were planned and acquired orthogonal to the main pulmonary artery 2 cm above the valve.

Black blood imaging of the pulmonary arteries was performed using a double inversion recovery fast spin echo sequence (DIR-FSE) using an 8 channel cardiac coil during a breath-hold. A stack of 8 mm slices with 10 mm spacing are taken in the axial orientation 4–6 slices through the pulmonary artery were acquired. There is a 180° nonselective inversion pulse, immediately followed by a slice-selective 180° de-inversion pulse. The TI is then calculated based on the R-R interval on the ECG, it is 650 msec at 60bpm. The other sequence parameters were: TR around 1000 msec (dependent upon R-R interval), TE 40 ms, FSE flip angle 90°, echo train length 32 with approximately 2.5 msec between echoes, field of view 4 cm, 256 × 256 matrix, 31.2 kHz bandwidth, ASSET parallel imaging factor 2.

Table E1: Training Cohort Correlations with mPAP and PVR and Area under the ROC Curve for the Diagnosis of PH, in Descending Order of Diagnostic Accuracy

	mPAP	PVR	AUC
Black blood score	0.694*	0.654	0.909
IV Septal angle (systole)	0.740*	0.704*	0.908
Systolic VMI	0.553*	0.563*	0.897
Diastolic PA area	0.504*	0.342*	0.878
Diastolic VMI	0.529*	0.533*	0.872
RV mass	0.535*	0.138*	0.862
RVESVI	0.585*	0.611*	0.854
RVEF	−0.602*	−0.623*	0.835
Av PA velocity	−0.505*	−0.548*	0.828
Systolic PA area	0.411*	0.234*	0.817
LVSVI	−0.491*	−0.597*	0.805
IV Septal angle (diastole)	0.548*	0.530*	0.794
RVEDVI	0.480*	0.480*	0.782
LVEDVI	−0.396*	−0.502*	0.738
LVEF	−0.302*	−0.368*	0.703

PA RAC	−0.357*	−0.382*	0.664
Aortic flow	−0.290*	−0.470*	0.653
4Ch LA area	−0.254*	−0.380*	0.643
2Ch LA length	0.102	−0.032	0.608
LV systolic mass	−0.145	−0.336*	0.598
PA flow	−0.138	−0.330*	0.586
RVSVI	−0.101	−0.159*	0.573
2Ch LA area	0.04	−0.123	0.566
LA volume index	−0.183*	−0.277*	0.552
4Ch LA length	−0.038	−0.115	0.535
LVESVI	−0.091	−0.107	0.531
LV diastolic mass	−0.031	−0.227*	0.512

mPAP = mean pulmonary arterial pressure, PVR = pulmonary vascular resistance, AUC = area under the ROC curve, RVEDV = right ventricular end-diastolic volume, RVESV = right ventricular end-systolic volume, RVEF = right ventricular ejection fraction, RV = right ventricle, LVEDV = left ventricular end-diastolic volume, LVESV = left ventricular end-systolic volume, LVEF = left ventricular ejection fraction, PA RAC = pulmonary artery relative area change, VMI = ventricular mass index, IVS = interventricular septum.

* $P < .001$.
